# Prognostic Factors of IDH Wild-Type Glioblastoma After Extensive Surgery: A Multimodal Atlas of Tumor Locations, Recurrences and Management

**DOI:** 10.3390/cancers18010063

**Published:** 2025-12-24

**Authors:** Hajar Selhane, Tiphaine Obara, Guillaume Vogin, René Anxionnat, Guillaume Gauchotte, Luc Taillandier, Marie Blonski, Fabien Rech

**Affiliations:** 1 Université de Lorraine, CHRU-Nancy, Service de Neurologie, F-54000 Nancy, France; 2 Université de Lorraine, CNRS, CRAN, F-54000 Nancy, France; 3 Centre François Baclesse, B.P 436, L-4005 Esch-sur-Alzette, Luxembourg; 4 Luxembourg Institute of Health, Department of Cancer Research, L-1210 Luxembourg, Luxembourg; 5 Université de Luxembourg, Faculté des Sciences, de Technologies et de Médecine, L-4364 Esch-Sur-Alzette, Luxembourg; 6 CHRU-Nancy, Université de Lorraine, Service de Neuroradiologie, F-54000 Nancy, France; 7 Université de Lorraine, IADI, INSERM U1254, F-54000 Nancy, France; 8 CHRU-Nancy, Université de Lorraine, Service d’Anatomopathologie, F-54000 Nancy, France; 9 Centre de Ressources Biologiques, CHRU-Nancy, F-54000 Nancy, France; 10 INSERM U1256, NGERE, F-54500 Nancy, France; 11 CHRU-Nancy, Université de Lorraine, Service de Neurochirurgie, F-54000 Nancy, France

**Keywords:** glioblastoma, surgery, MRI atlas, recurrence

## Abstract

Introduction: Glioblastomas have poor prognosis despite aggressive treatment. Patterns of recurrence and overall survival (OS) can be very different. The population with complete resection having a so-called good prognosis can nevertheless present poor OS. Our purpose was to assess the OS and patterns of recurrence thanks to multimodal statistical maps in glioblastoma with large extent of resection (residue < 10 mL). Methods: adult patients presenting IDH wild-type glioblastoma between 2013 and 2019 were selected. Clinical data and MRI characteristics were collected. Preoperative, postoperative, and recurrence volumes were segmented and normalized in the MNI space to compute statistical maps. Log-rank test and Cox model were used to assess OS and prognosis factors. Results: 60 patients were included. Mean residual volume was 0.89 ± 2 mL. Median OS was 22.3 months (95% CI: (20–35)). Initial location in the corpus callosum was associated with low OS (317 vs. 783 days, HR = 0.46, *p* = 0.003). At recurrence, KPS > 90 and tumor volume < 10 mL were associated with higher OS (*p* =0.006 and *p* = 0.05). Tumor contact with the SVZ as well as multifocal recurrence did not show any impact on the OS. Conclusions: High OS can be obtained thanks to surgery with residual volume < 10 mL. Invasion of the corpus callosum at diagnosis is associated with a poor prognosis despite a large extent of resection. Results suggest that large resection near the SVZ might decrease its putative influence on OS.

## Simple Summary

Glioblastoma is one of the most aggressive tumors of the brain despite complete resection of the contrast enhancement. The aim of our retrospective study was to identify the pattern of recurrence after complete resection. We showed that in such a homogeneous group of patients, overall survival might be low according to initial (corpus callosum) and recurrence location (left atrium). Multifocal recurrences seemed to be correlated with tumor location (corpus callosum, frontal left, and temporal bilateral) whereas relationship between the subventricular zone and overall survival seemed to be more complex and might depend on the extent of resection and tumor location. We also showed that even in complete resection, 92% of recurrence occurred in the vicinity of the postoperative cavity. These findings also suggest identifying new strategies to improve the local control of the disease even after large surgery.

## 1. Introduction

Glioblastoma is the most common malignant brain tumor in adults. Prognosis is poor despite aggressive treatment by surgery and radiochemotherapy [1,2,3]. Despite an abundant literature concerning prognostic factors, populations are often heterogeneous or lack radiological data [4,5,6,7,8,9]. Then, assessing the link between topography and survival or recurrence is difficult and identifying the evolution of specific subsets of patients according to the first-line treatment is almost impossible. However, despite differences between teams, overall therapeutic decisions are usually similar, especially concerning the decision to perform biopsy or surgery at diagnosis [10,11]. Consequently, it would be preferable to determine prognostic factors after a homogeneous first-line treatment, particularly if predictable, rather than comparing multiple modalities or entities. Such information would be more helpful to predict evolution at an individual level.

It is noteworthy that patients benefiting from extensive surgery could present poor prognosis with multifocal recurrence, although complete resection is considered as a good prognostic factor [9,12]. The relationship between the tumor and the subventricular zone (SVZ), via the existence of tumor progenitor cells, has been proposed to explain such poor evolution, including after gross total resection [6,7,13]. Consequently, our objective was to assess the correlation between clinical data, overall survival, tumor initial topography, and pattern of recurrence on original statistical maps of a homogeneous population of IDH wild-type glioblastoma who underwent a complete or sub-complete resection defined by a residual volume inferior to 10 mL [12].

## 2. Patients and Methods

Consecutive adult patients treated for an IDH wild-type glioblastoma between 2013 and 2019 were identified in our center. Inclusion criteria were age > 18 years old, IDH wild-type glioblastoma according to the World Health Organization 2021 classification [14], unique supratentorial location, total/subtotal resection (postoperative residue < 10 mL [12]), adjuvant treatment by radiochemotherapy according to Stupp et al. [2], at least one site of first recurrence under the form of a contrast enhancement after beginning the adjuvant therapy, and MRI available at preoperative, postoperative, and recurrence times. Informed consent was obtained before surgery.

Clinical data such as Karnofsky Performance Status (KPS), age, sex, adjuvant therapies, overall survival (OS), and progression-free survival (PFS), as well as radiological characteristics such as tumor volume and location, were collected retrospectively from our database. Contrast-enhancement 3D-T1 weighted MRI were acquired for each patient during preoperative and postoperative (<72 h) time and at recurrence.

Segmentations of the preoperative tumor, residue, and recurrence were performed using ITK-SNAP v4.0.1 software (www.itksnap.org) [15] by AS and reviewed by FR. All segmentations were performed on the contrast-enhancement tumor and necrotic components then registered as Nifti binary masks. Masks were normalized in the MNI space using SPM12 (http://www.fil.ion.ucl.ac.uk/spm/software/spm12/), implemented in the Matlab environment (version R2016a, The Mathworks, Inc., Natick, MA, USA) as previously described [16]. Recurrence was defined as the first apparition of the contrast enhancement which was immediately or later considered as a relapse according to the assessment of the entire MRI follow-up and medical decision during the multidisciplinary tumor board. Therefore, we considered a contrast enhancement as a relapse only if a second-line treatment was decided for it. Statistical maps were computed using an in-house routine in Python 3.12 (using Numpy and Nilearn packages [17,18]). Masks were overlapped to compute tumor frequency or mean of each variable of interest according to groups emerging from statistical analysis. MRIcroGL v1.2.20220720 (http://www.mccauslandcenter.sc.edu/mricrogl/) was used to perform color maps and visual comparison between subgroups.

All data analyses were performed using R version 4.0. For descriptive statistics, we used numbers and percentages; for qualitative ones, variables; and for quantitative ones, medians and ranges. OS and PFS were calculated from the time of surgical resection until death and progression, respectively. Survival curves were estimated using the Kaplan–Meier method with a 95% confidence interval and differences tested by the log-rank test. To assess prognostic factors, univariate analysis was first performed using log-rank tests for qualitative variables and univariate Cox models for quantitative ones. Factors with *p*-value ≤ 0.05 were considered statistically significant.

## 3. Results

### 3.1. Patients’ Characteristics and Management at Diagnosis

Sixty patients were included in the study. Clinical and tumor characteristics are detailed in Table 1.

**Figure 1:**
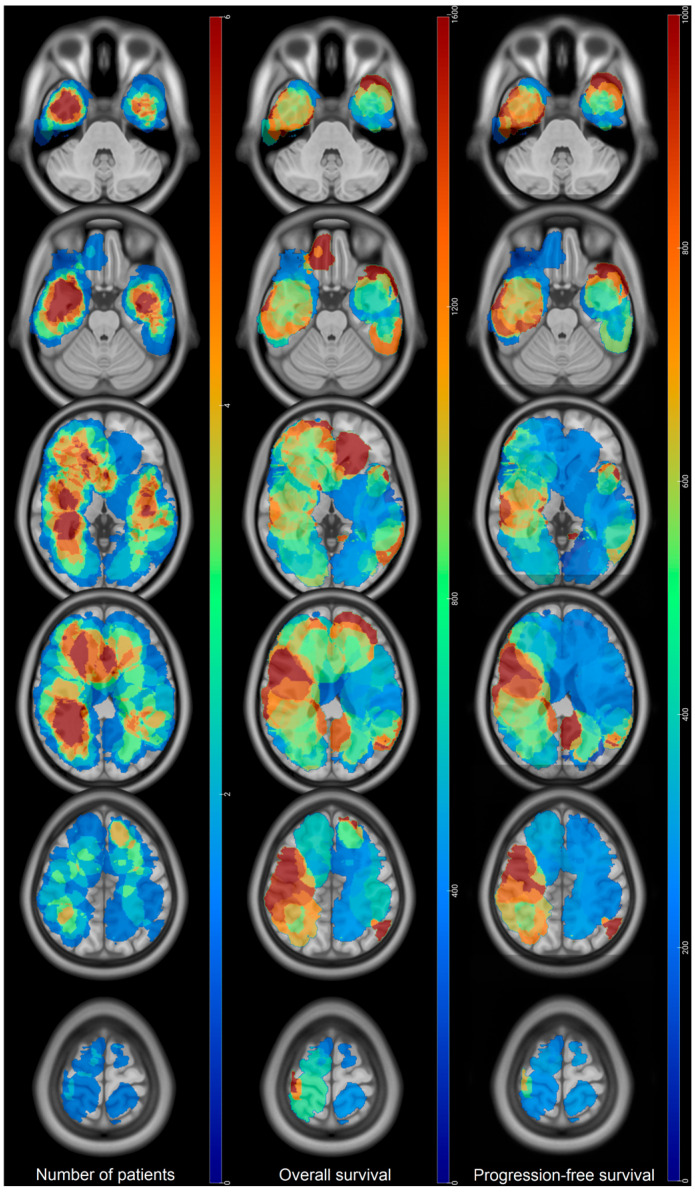
Maps of tumors at diagnosis displayed on the ICMB152 asymmetrical template. OS and PFS are expressed in days. Scales are different. **Numberof patients:** red regions correspond to the highest number of patients (*n* = 6) for a specific tumor location at diagnosis, and blue ones are the lowest ones. **Overall survival:** red regions correspond to locations where overall survival (in days) is the longest (1600 days) and blue ones correspond to the shortest OS. **Progression-free survival:** red regions correspond to locations where PFS is the longest (1000 days) and blue ones correspond to the shortest PFS.

Mean age at diagnosis was 61 ± 10 and sex-ratio was 1.4 (M/F). Mean KPS at diagnosis and postoperative time were, respectively, 81 ± 9 and 82 ± 9. Most tumors were located on the right hemisphere (52%). Frontal (26%) and temporal (38%) lobes represented main locations (Figure 1). 38% were periventricular and 12% invaded the corpus callosum.

**Figure 2:**
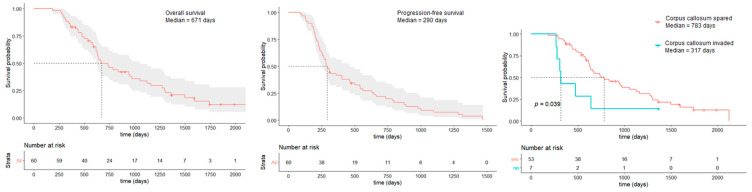
Kaplan–Meier estimates of the OS and PFS of the whole cohort and according to the invasion of the corpus callosum.

Median OS was estimated at 671 days (95% CI: 610–1056) and median PFS was estimated at 290 days (95% CI: 256–465) (Figure 2). OS > 24 months were mainly observed in the frontobasal location and on the right hemisphere, especially in the right temporal lobe and the right atrium. Premotor tumors did not show OS > 24 months. Tumors located in the subventricular zone had shorter median OS (540 days) than non-SVZ one (660 days), although non significative (*p* = 0.3).

Location in the corpus callosum at diagnosis was associated with shorter OS in multivariate analysis (median OS = 317 days when corpus callosum was invaded, 783 days when it was safe, hazard ratio = 0.46, *p* = 0.003) (Table 2) despite very low residual volumes (five complete resections, two residual volumes < 0.5 mL). Mean KPS at diagnosis for those tumors was 79 ± 11. Only three of these patients benefited from a complete STUPP protocol because recurrences occurred in four patients between 2 and 5 months after the end of the radiotherapy and during the adjuvant chemotherapy.

Resections of the contrast enhancement were complete in 36 patients (60%); residual volumes were between 0 and 5 mL in 19 patients (31.6%) and between 5 and 10 mL in 5 patients (8.3%), which corresponded to resection > 98% of the preoperative volume in 51 patients (85%), between 95 and 98% in 2 patients (3.3%), and between 85 and 95% in 7 patients (11.7%). Mean percent of resection of contrast enhancement for the tumor invading the corpus callosum was 99.9%.

Thirty-two patients (52.3%) received a complete treatment of adjuvant radiochemotherapy (30 sessions of radiotherapy with concomitant temozolomide followed by six cycles of temozolomide [2]), which was associated with a longer OS in univariate analysis (median OS for complete STUPP protocol = 924 days vs. 457 days for incomplete protocol, *p* = 0.01). Fourteen patients (23.3%) received more than six cycles of temozolomide, which was associated with a longer OS in univariate analysis (median OS = 826 days vs. 605 days, *p* = 0.01) (Table 2).

### 3.2. Patients’ Characteristics and Management at Recurrence

**Figure 3:**
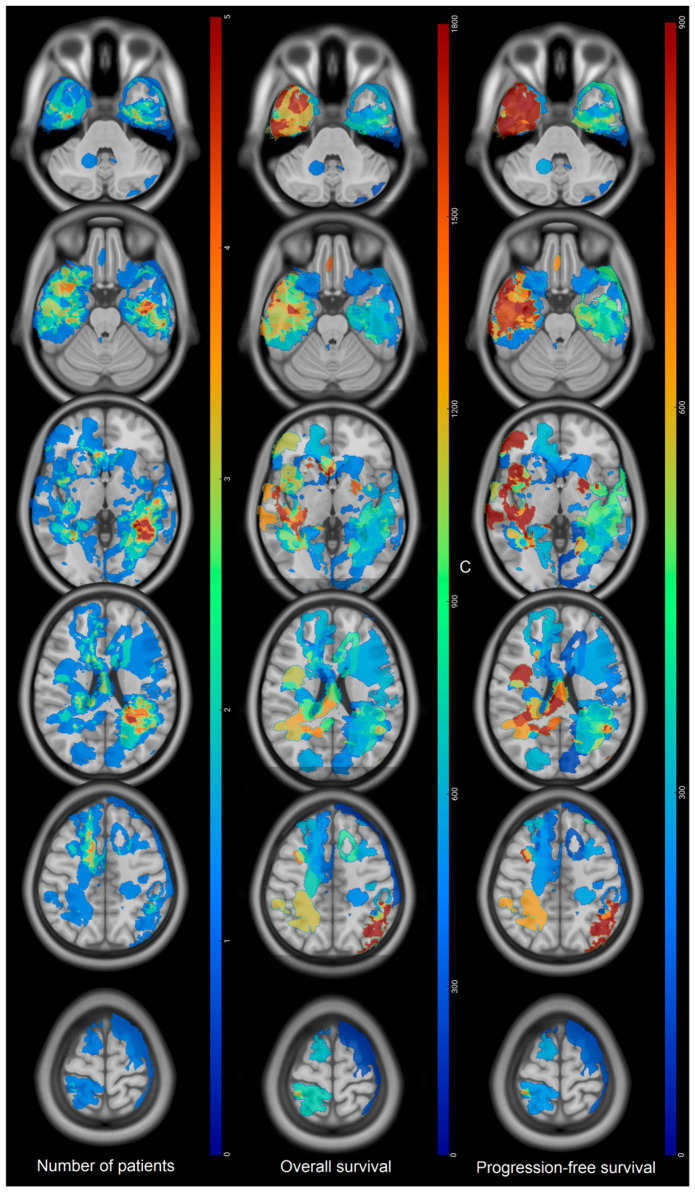
Maps of tumors at recurrence displayed on the ICMB152 asymmetrical template. OS and PFS are expressed in days. Scales are different. **Number of patients:** red regions show the highest number of recurrences (*n* = 5) while blue ones show the lowest number of recurrences. **Overall survival:** red regions correspond to recurrences locations where overall survival (in days) is the longest (1800 days) and blue ones correspond to the shortest OS. **Progression-free survival:** red regions correspond to recurrences locations with the longest PFS (900 days) and blue ones correspond to the shortest PFS.

**Figure 4:**
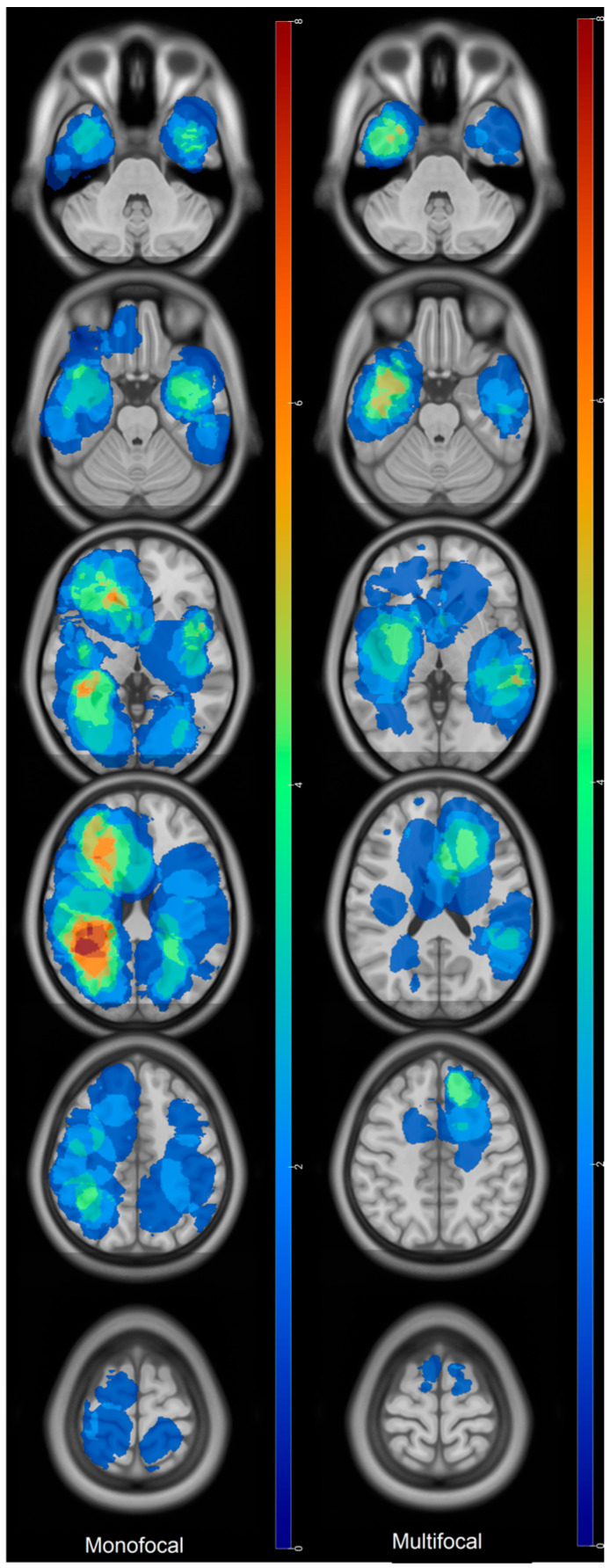
Origins of monofocal and multifocal recurrences. Colors bars indicate the number of patients and are identical. **Monofocal:** red regions correspond to the most frequent (*n* = 8) location of the primary tumor giving monofocal recurrence. Blue ones are the least frequent. **Multifocal:** red regions correspond to the most frequent (*n* = 8) location of the primary tumor giving multifocal recurrence. Blue ones are the least frequent.

Recurrences occurred on the margin of the postoperative cavity in 92% of the cases. Thirty-three percent of the recurrences were also distant from the initial site of the tumor and multifocal. Eighty-one percent (30/37) of tumors that were not in the SVZ at diagnosis showed recurrence in the SVZ. Main sites of recurrence were in the temporal lobe and around the atrium of the lateral ventricles (Figure 3). Multifocal recurrences came mainly from temporal (bilateral) and left frontal tumors (Figure 4). Monofocal recurrences tend to occur later (median PFS = 311 days vs. 268 days for multifocal) and show longer OS (median OS = 666 days vs. 444 days for multifocal, *p* = 0.1) (Table 2). Periventricular and left tumors seemed to occur more quickly according to Figure 1. Mean recurrence volume was 19 ± 18 mL and a recurrence volume < 10 mL was associated with longer OS in univariate analysis (*p* = 0.05) (Table 2).

**Figure 5:**
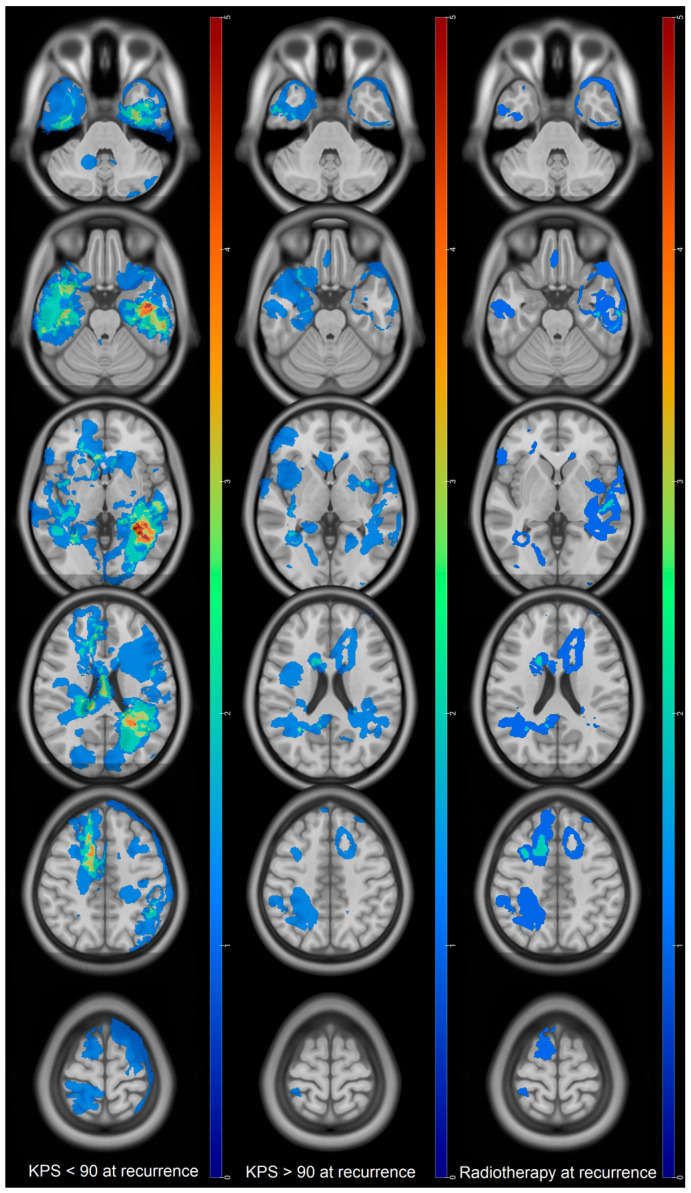
Maps of recurrence showing KPS < 90 and >90 and recurrences benefiting from radiotherapy. Colors bars indicate the number of patients and are identical to allow comparison. **KPS < 90 at recurrence:** red regions indicate where recurrences in patients with KPS < 90 are the most frequent (*n* = 5) while blue zones are the least frequent. **KPS > 90 at recurrence:** red regions are locations where recurrences for patients with KPS > 90 are the most frequent (*n* = 5) while blue zones are the least frequent. **Radiotherapy at recurrence:** red regions are locations of tumors that are most likely to benefit from radiotherapy while blue regions are less likely.

Mean KPS at recurrence was 80 ± 12. KPS ≥ 90 at recurrence was associated with a longer OS in univariate analysis (972 days vs. 538 days for KPS < 90, *p* = 0.006) (Table 2). Recurrences associated with a low KPS were mainly located in the left temporal lobe, the atrium of the left lateral ventricle, the periventricular zone, the right cingulate gyrus, and the posterior fossa (Figure 5). Twenty-four percent of the high IK recurrence were multifocal versus 40% for low IK recurrence. Pre-STUPP mean IK was 78.8 for low IK recurrence versus 88 for high IK recurrence.

**Figure 6:**
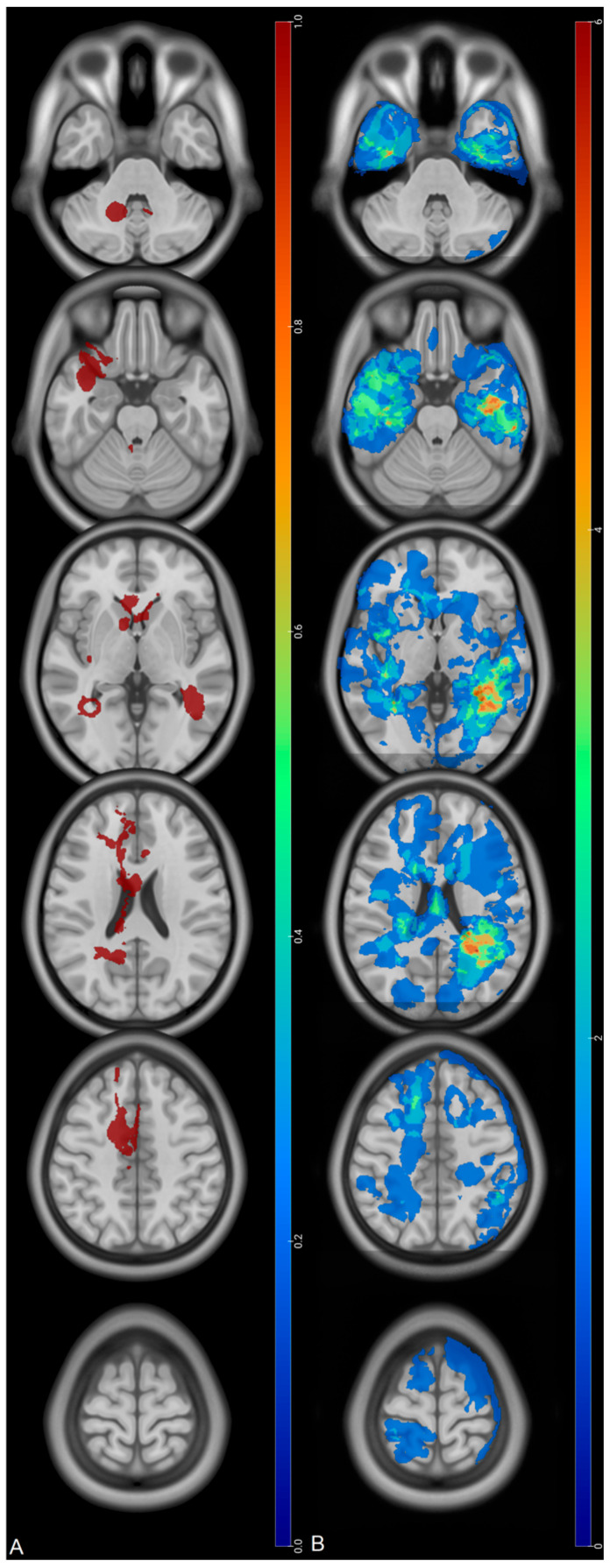
Maps of recurrence according to the corpus callosum invasion. (**A**): Recurrences of tumors invading the corpus callosum at diagnosis (only one recurrence per voxel). (**B**): Recurrences of tumors sparing the corpus callosum at diagnosis. Red regions correspond to the highest number of recurrences (*n* = 6) while blue ones correspond to the lowest number. Scales are different.

Recurrences of tumors invading the corpus callosum at diagnosis (*n* =7) were mainly periventricular and, among them, three became multifocal (Figure 6). Interestingly, the mean KPS at recurrence for those tumors was 70. Mean delay of PFS for those tumors were 331± 253 days.

Seventeen patients (28%) benefited from radiotherapy at recurrence. Median OS was 906 days whereas median OS in the group without radiotherapy was 616 days (*p* = 0.06) (Table 2, Figure 5).

## 4. Discussion

This is the first study assessing the pattern of recurrence in a population of IDH wild-type glioblastoma which benefited from total or subtotal resections (gross total resection and/or residue < 10 mL). Most studies reporting gliomas grouped grade III or IV astrocytoma or patients with very different management such as biopsy and surgery or adjuvant treatment in the pre-STUPP era [2,4,5,9,19]. In this work, we precisely assessed the pattern of recurrence in the most homogeneous possible population of patients who benefited from the optimal and standard care in a glioblastoma at diagnosis to understand which factors might influence the prognosis.

Our population did not differ from others surgical series. Median age was 61 years old, males were more represented than females, and tumor locations were quite similar [4,5,12,20]. Median PFS was estimated at 290 days (9.6 months) and was longer than described by Roux et al. [4] (7.5 months) but shorter than Chen et al. [20] (10.4 months). Median OS was estimated at 671 days (22.3 months) and mean OS at 976 ± 240 days, which was higher than the previous reports compiling results from several cohorts [12,19,20]. These differences are likely due to our decision to select a large extent of resections with residual volume inferior to 10 mL, a cut-off defined according to Ellingson et al. [12]. This explains the better OS and how we succeeded in harmonizing our cohort and decreased the influence of resection on OS. Moreover, age and KPS at diagnosis were not correlated with OS likely because patients were operable and then improved by surgery if they presented neurological impairment.

Moreover, the OS map showed longer OS for tumors located in the right hemisphere, especially in the temporal lobe and the right atrium, for frontobasal and left frontal anterior tumors in accordance with Roux and Fyllingen et al. [4,5] despite how no voxel-lesion symptom mapping analysis could be performed. Shorter OS were also observed for left hemispheric tumors, especially temporal and occipital ones. Such results cannot be explained by a lesser extent of resections in those locations as reported in the brain maps of tumor resection [10] because only patients with residual tumor volume < 10 mL were selected. Nevertheless, it is possible that the extent of resections beyond the contrast enhancement were lesser than in the right hemisphere for functional purposes, explaining those shorter OS [19,21]. Given the fact that KPS at recurrence was associated with OS in univariate analysis, another potential explanation might be that the alteration of KPS by focal recurrence in the left eloquent area, such as the temporal lobe and atrium, impacts the OS (Figure 5).

One major finding related to tumor location is that the initial location on the corpus callosum was associated with a lower OS in multivariate analysis. Such results are in accordance with Fyllingen et al. who assumed that proximity to the center of the third ventricle was associated with lower OS [5]. Recurrences associated with this location were in the SVZ, near the subarachnoid space, or in the posterior fossa, contrary to tumors sparing the corpus callosum whose recurrence, despite periventricular location too, were also focal, more cortical, and distributed in the brain (Figure 6). However, recurrences of half of the tumors invading the corpus callosum were focal, involving the SZV in the neighborhood, thus excluding a CSF dissemination at least at the first recurrence.

Many studies reported that tumors located near or in the SVZ have bad prognosis, likely because of specific histological pattern or tumor progenitor cells located in the SVZ [6,7,13,22,23,24,25]. We did not perform specific histological or molecular analysis and our results did not show an involvement of the SVZ in the prognosis, but such opposite results have already been mentioned by Chen et al. [26]. We also noted that recurrences tend to occur near the SVZ but, as we performed large extent of resections usually up to the minimal common brain [27], the remaining brain tissue was often central, near the SVZ. It should also be kept in mind that the definition of the SVZ remains unclear between studies, ranging from 0 to 1 cm [6,7,13,20,26]. Moreover, those studies reported a low extent of resections for tumors invading the SVZ and did not specify the location of SVZ invasion, namely temporal or frontal lobes. Here, we selected tumors with a large extent of resections which could have modified the impact of SVZ invasion on OS. Temporal tumors also affected the SVZ frequently but did not show low OS in our study. One explanation is that SVZ progenitor cells might have been removed by a large extent of resections of the ventricle wall in temporal locations, whereas it was more difficult for tumors invading the genu of the corpus callosum due to the proximity of the basal ganglia, as proposed by Saito et al. [28]. Another possible explanation is that dissemination in the corpus callosum occurs along white matter pathways [29,30]. Indeed, despite almost complete resections of the contrast enhancement in most of the cases of corpus callosum involvement, it is likely that contralateral tumoral infiltration, especially under the form of the FLAIR signal, was already present and had affected the prognosis even after the first recurrence [19,21].

Multifocal recurrence pattern was associated with a lower OS, despite not being significative. Nevertheless, it is noteworthy that Adeberg et al. did not show any correlation between long-term and short-term survivals in terms of multifocal disease [22]. Initial tumor locations of such multifocal recurrences were mainly frontal left, on the corpus callosum and temporal bilateral. We presumed that less extent of resection of the FLAIR component in left tumors as well as less resection of the SVZ in some temporal tumors might be the cause of such dissemination, as previously proposed for the corpus callosum.

However, 92% of the recurrences occurred also around the postoperative cavity despite large extent of resection and adjuvant therapy. This is consistent with previous reports showing recurrences within the 2 cm of the tumor border [8,9] and pleads to improve the local control of the disease thanks to new focal therapies [31,32,33]. In addition, given that SVZ proximity did not impact the OS, this does not plead for irradiation of the SVZ after surgery, an option which still remains controversial [26,34,35].

Volume at recurrence > 10 mL was associated with low OS in univariate analysis. This might signify that tumoral quantity is more important than its topography, namely if it is focal or multifocal [36]. Interestingly, we took the first occurrence of a contrast enhancement as recurrence, so, as follow-ups were almost the same for each patient, we presume that this could represent the capacity of the tumor to replicate more quickly between two MRI, which could be correlated to its aggressiveness. One other possible explanation is that treatment at recurrence occurred at amore advanced level of the disease and was, then, less effective.

Radiotherapy at recurrence and KPS > 90 showed a relationship with longer OS in univariate analysis. It has already been shown that decreasing KPS at recurrence was predictive of low OS whereas surgical resection and chemotherapy were good prognosis factors [37]. Radiotherapy at recurrence has also shown some interest in previous reports [36,38,39]. The New Combs Prognostic Score [36] for reirradiation of glioma also includes the KPS at recurrence. These two factors are likely linked together as shown for the maps of RT at recurrence and high KPS which can almost be overlapped (Figure 5). Moreover, radiotherapy, as surgery, is a focal treatment and might reflect that tumor evolution is focal and that the patient is in good enough condition to undergo the treatment. To our view, this seems to suggest that radiotherapy can be rather efficient at recurrence on highly selected patients.

Thanks to probabilistic maps, Müller et al. showed that decisions between biopsy or surgery for glioblastoma are usually consensual between teams [11] and that the extent of resections were almost the same, except for the right anterior limb of the internal capsule and the right caudate nucleus [10]. This highlights two crucial points: Firstly, resection of tumor in contact with the SVZ at the level of the genu of the corpus callosum presents some difference between teams which might affect the prognosis, according to Saito et al., and might explain differential results related to the SVZ [28]. Secondly, the degree of extent of resection can be precisely predictable given those probabilistic maps, especially for expert neurosurgical teams. Such predictability of the extent of resection can then be used to apprehend overall survival at diagnosis time for a specific surgical population, which is precisely the strength of our work. Indeed, we reported here all the residual volumes and the extent of resections instead of the unclear gross total/subtotal resection distinction and removed all the IDH mutants or grade III/IV foci which have totally different prognosis [40]. Consequently, we provide clinical and radiological data related to the recurrence in a population of de novo glioblastoma operated with large extent of resection, which might be helpful for neuro-onco-surgical teams to apprehend a patient’s prognosis as soon as the surgical decision of resection is taken. It emphasizes the role of the corpus callosum and brings new elements about the role of the SVZ in the prognosis, which needs to be explored in future studies with a larger population. It also highlights the lack of local control of the disease with 92% of recurrences occurring in the irradiation field, despite the large extent of resection. Taken together, these results suggest the intensification of treatments for a specific subset of patients, thanks to earlier chemotherapy or adjuvant focal treatment [31,32,33,41].

## 5. Limits

Our results might differ from previous studies as our population benefited from total or subtotal resection (residue < 10 mL). This led to a highly selected population of patients. A lack of power due to the small number of patients might explain why only the corpus callosum invasion showed statistical significance in the multivariate model. Therefore, results and conclusions should not be extrapolated to the whole population of glioblastoma wild-type.

Moreover, recurrence was defined by the occurrence of contrast enhancement without considering the FLAIR signal. Post-recurrence evolution might then differ from the literature, but it is noteworthy that patients with FLAIR progression will not have the same treatment as those with contrast-enhancing tumors. In addition, we did not take account of the second recurrence, thus treatment at this time might have impacted the survival.

Although maps of tumor location can be biased by the mass effect, qualitative aspects of the location such as SVZ or corpus callosum invasion were defined visually by experts for each patient. Moreover, tumor recurrence volumes were too small to perform voxel-lesion symptom mapping, leading us to perform only a descriptive analysis of the map.

## 6. Conclusions

We reported here a unique series of patients operated on for an IDH wild-type glioblastoma with large extent of resections. Tumors located near the SVZ did not show lower OS whereas invasion of the corpus callosum was correlated with shorter OS. KPS > 90 at recurrence confirmed its association with longer OS. These results might be helpful to identify patients with potential poor prognosis after large surgery and plead for a more aggressive treatment for those tumor locations. Studies with a larger cohort are mandatory to assess, particularly, the impact of multifocal recurrences and radiotherapy at relapse on overall survival.

## Figures and Tables

**Table 1:** Characteristics of patients and tumors.

Parameters	Value
**Age in years (mean ± standard deviation)**	61 ± 10
**Sex**	
Female	25 (42%)
Male	35 (58%)
**Volume in mL (mean ± standard deviation)**	
Initial	56 ± 36
Residue	0.89 ± 2
Recurrence	19 ± 18
Extent of resection (%)	98.7 ± 3
**Tumor location at diagnosis *n* (%)**	
SVZ (<5 mm to the ventricle wall)	23 (38%)
Corpus Callosum	7 (12%)
Inner Temporal	4 (7%)
Cortical	52 (85%)
Subcortical ^1^	3 (5%)
**Tumor’s side at diagnosis *n* (%)**	
Right	31 (52%)
Left	26 (42%)
Bilateral	3 (5%)
**Topography at diagnosis *n* (%)**	
Frontal	16 (26%)
Temporal	23 (38%)
Occipital	2 (3%)
Parietal	2 (3%)
Occipito-temporal	1 (2%)
Temporo-occipital	6 (10%)
Parieto-occipital	6 (10%)
Fronto-parietal	4 (7%)
**KPS (mean ± standard deviation)**	
Diagnosis	81 ± 9
Recurrence	80 ± 12
**STUPP protocol**	
Complete	32 (53%)
Incomplete	28 (47%)
Extended beyond 6 months	14 (23%)
**Tumor location at relapse *n* (%)**	
Postoperative cavity	55 (92%)
SVZ ^2^	30 (50%)
Cortex	44 (73%)
Corpus Callosum ^3^	14 (23%)
Multifocal	20 (33%)
Distant ^4^	20 (33%)
Infratentorial	4 (7%)
**Treatment at relapse *n* (%)**	
Radiotherapy	17(28%)
Chemotherapy	50 (83%)
Surgery	4 (7%)
Palliative	4(7%)
**Median OS (95% CI)**	671 days (610–1056)
	22.3 months (20–35)
**Median PFS (95% CI)**	290 days (256–465)
	9.6 months (8.5–15.5)

^1^ Tumor sparing cortex and SVZ. ^2^ Excluding tumor invading SVZ at diagnosis. ^3^ Excluding tumor invading the corpus callosum at diagnosis. ^4^ Recurrence located beyond 2 cm of the border of the postoperative cavity.

**Table 2:** Prognostic factors in univariate and multivariate analysis.

	Univariate	Multivariate		
	*p*-Value	*p*-Value	Hazard Ratio	Median OS
Age < 60	0.3			
KPS at diagnosis < 90	0.4			
Topography	0.4			
SVZ contact	0.3			
Corpus callosum invasion	**0.04**	**0.003**	**1** **0.46**	**317** **783**
Preoperative KPS < 90	0.3			
Postoperative KPS < 90	0.2			
Pre-STUPP KPS < 90	0.09			
Complete STUPP	**0.01**	0.77		
STUPP beyond 6 months	**0.01**	0.22		
KPS at recurrence < 90	**0.006**	0.76		
Recurrence volume < 10 mL	**0.05**	0.06		
Recurrence in corpus callosum	0.8			
Multifocal recurrence	0.1			
Distant recurrence	0.5			
Radiotherapy at recurrence	0.06	0.69		
Chemotherapy at recurrence	0.7			

Bold values indicate significant *p* values (≤ 0.05).

## Data Availability

Data available on request due to legal restrictions.
